# Association between Obesity and Short-Term Patient-Reported Outcomes following Total Knee Arthroplasty: A Retrospective Cohort Study in Japan

**DOI:** 10.3390/jcm13051291

**Published:** 2024-02-24

**Authors:** Ryu Ishimoto, Hirotaka Mutsuzaki, Yukiyo Shimizu, Kenichi Yoshikawa, Kazunori Koseki, Ryoko Takeuchi, Shuji Matsumoto, Yasushi Hada

**Affiliations:** 1 Graduate School of Comprehensive Human Sciences, University of Tsukuba, Tsukuba 305-8575, Japan; ishimotori@ipu.ac.jp; 2 Department of Rehabilitation Medicine, Ibaraki Prefectural University of Health Sciences Hospital, Ami 300-0331, Japan; 3 Center for Medical Science, Ibaraki Prefectural University of Health Sciences, Ami 300-0394, Japan; 4 Department of Orthopaedic Surgery, Ibaraki Prefectural University of Health Sciences Hospital, Ami 300-0331, Japan; 5 Department of Rehabilitation Medicine, Institute of Medicine, University of Tsukuba, Tsukuba 305-8575, Japan; 6 Department of Physical Therapy, Ibaraki Prefectural University of Health Sciences Hospital, Ami 300-0331, Japan

**Keywords:** total knee arthroplasty (TKA), obesity, patient-reported outcomes, Western Ontario and McMaster Universities Osteoarthritis Index (WOMAC), inpatient rehabilitation

## Abstract

**Background:** This study investigated the association between obesity and short-term patient-reported outcomes after total knee arthroplasty (TKA). **Methods:** The primary outcomes were the Western Ontario and McMaster Universities Osteoarthritis Index’s (WOMAC) pain and function scores. Data were collected preoperatively and 2 and 4 weeks after surgery. Patients were stratified into three groups based on body mass index (BMI): normal weight (BMI < 24.99 kg/m^2^), overweight (25 ≤ BMI < 29.99 kg/m^2^), and obese (BMI ≥ 30 kg/m^2^). The associations between BMI and the WOMAC pain and function scores were assessed using generalized linear mixed models. **Results:** Among the 102 patients (median age: 75.0, women [85.3%]), 29.4%, 48.0%, and 22.5% were normal weight, overweight, and obese, respectively. The mean pain and function scores at baseline were similar across the BMI-stratified groups (*p* = 0.727 and 0.277, respectively). The pain score significantly improved 2 weeks post-surgery (*p* = 0.001). The function score improved significantly 4 weeks post-surgery (*p* < 0.001). The group and group-by-time interaction effects lacked statistical significance. **Conclusions:** All patients statistically and clinically showed relevant pain reduction and functional improvement shortly after TKA, irrespective of their obesity status. These data may help healthcare professionals discuss the expectations of pain amelioration and functional improvement with TKA candidates.

## 1. Introduction

Osteoarthritis (OA) is a chronic degenerative disease that causes pain, functional limitations, and deterioration in quality of life [1,2]. The knee is one of the most affected joints; the prevalence of knee OA is estimated to be 7–54.6% [3,4,5,6,7,8]. This prevalence increases with age, showing a greater female predilection [2]. Furthermore, obesity has been established as a risk factor for knee OA [2]. The incidence of obesity in patients with advanced knee OA is expected to rise because of the aging population and increasing prevalence of obesity, resulting in a high disease burden and implications for patients and healthcare systems [2,9,10,11,12].

Total knee arthroplasty (TKA) is a well-recognized and effective surgical intervention in patients with advanced OA that relieves pain, revives function, and improves quality of life [13,14]. Functional measures, such as knee range of motion, quadriceps strength, and gait speed, reportedly decline shortly after surgery, followed by substantial improvements in the first few months [15,16,17,18,19,20]. However, approximately 10–20% of patients were unsatisfied with TKA [21]. Objective outcomes may not always align with patients’ subjective perceptions [22], underscoring the significance of patient-reported outcome measures. Further information regarding early patient-reported outcome measures and factors influencing this recovery phase is crucial for optimizing early rehabilitation therapies and improving the quality of care and patient satisfaction after surgery [23].

Obesity has been determined as a factor influencing outcomes after TKA. Increased complication risks, including infections, thrombotic events, and implant loosening, have been widely reported in patients with obesity compared with those without obesity [24,25,26]. However, the effect of obesity on patient-reported outcomes remains unclear. Although some studies reported no association, others reported either negative or positive associations between obesity and postoperative pain and functional outcomes [16,18,19,20,23,26,27,28,29,30,31,32,33]. Previous studies have mainly focused on mid- to long-term outcomes, with limited evidence on the early recovery phase [30]. Furthermore, most studies investigating this association have been conducted in Western countries. Only a few studies have been conducted in Asian countries [32,33,34].

Recent studies have investigated the effects of obesity on short-term patient-reported functional outcomes. These studies have determined obesity as a risk factor for poor patient-reported outcomes at 6, 8, and 12 weeks after TKA [23,30]. However, these findings derived from studies comprising Western patients may not apply to their Japanese counterparts, wherein the prevalence of obesity has been reported to be lower than that in Western countries [10,35,36,37]. In addition, the length of stay in hospitals or rehabilitation centers after TKA varies between countries; it is much longer in Japan than in Western countries [38,39]. Additionally, patients in Japan commonly undergo inpatient rehabilitation during this period to improve their activities of daily living and walking ability [40]. Given that the frequency of knee arthroplasties is expected to rise over the next ten years in Japan [9], understanding the role of obesity in short-term patient-reported outcomes in Japanese patients is imperative. This understanding would enhance early recovery, patient satisfaction, and quality of care following TKA.

Therefore, this study aimed to investigate the association between obesity and short-term patient-reported outcomes following TKA. Based on previous studies [23,30], we hypothesized that patients with obesity would exhibit poorer short-term patient-reported pain and functional outcomes compared with those without obesity.

## 2. Materials and Methods

### 2.1. Participants and Setting

Patients admitted to our hospital for primary TKA between January 2016 and November 2023 were eligible for inclusion in this study. The participants completed the Western Ontario and McMaster Universities Osteoarthritis Index (WOMAC) before and after surgery, a standard procedure at our institution. Each unilateral TKA was counted as a separate entity in patients who underwent the procedure for both knees but at different time points. The exclusion criteria comprised patients with acute medical conditions that required the discontinuation of rehabilitation therapies, weight-bearing restrictions on the lower limbs, comorbid neurological dysfunction, simultaneous bilateral TKA, or missing baseline data.

**Figure 1:**
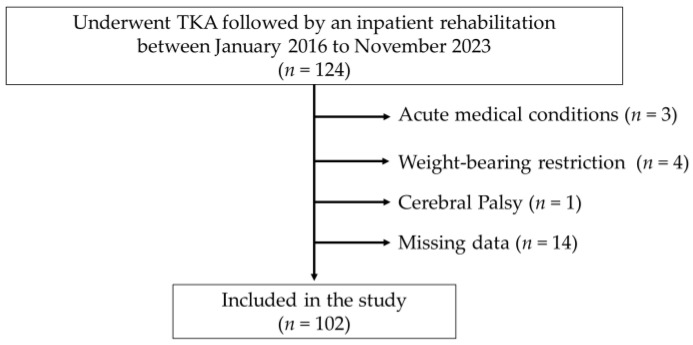
Recruitment flowchart.

The participant recruitment flowchart is depicted in Figure 1. During the study period, 124 patients underwent TKA. Three patients were transferred to other hospitals after surgery due to acute medical conditions (acute heart failure, electrolyte abnormality, and surgical site infection). In addition, the following patients were excluded: three patients requiring weight-bearing restrictions on the operated leg because of a patellar fracture, one requiring weight-bearing restrictions on the contralateral leg owing to a tibial fracture, and one with cerebral palsy. Furthermore, fourteen patients with missing baseline data were also excluded from the study. The remaining 102 patients were included in this study.

### 2.2. Surgery and Postoperative Rehabilitation

Prior to surgery, the patients received epidural analgesia with ropivacaine (0.375%). In cases where an epidural catheter insertion was not possible due to severe vertebral deformity or the presence of a screw or rod, femoral and sciatic nerve blocks were used instead with ropivacaine (0.375%). Under general anesthesia, a medial parapatellar approach was used to perform the surgery without replacing the patella. The surgery aimed to achieve mechanical alignment using an intramedullary alignment rod and an extramedullary guide system for femoral and tibial resection, respectively. The stability of ligament balance was confirmed after fixing the components. An intra-articular suction drain was placed prior to the closure of the joint capsule; it remained connected to a vacuum bag until it was removed on the first postoperative day. 

Clinical care pathways were implemented to standardize postoperative patient care, facilitate an interdisciplinary approach, and streamline the efforts of healthcare professionals responsible for patient care. Postoperative pain management included patient-controlled epidural analgesia or femoral nerve block with ropivacaine (0.16%) for 48 h following TKA. In addition, patients received an intravenous acetaminophen injection (1 g for BW ≥ 50 kg, 0.6 g for 40 < BW < 50 kg, and 0.3 g for BW < 40) every 6 h until postoperative day one. Subsequently, the patients received either loxoprofen sodium hydrate (60 mg) or acetaminophen (300 mg) three times a day, with the latter being prescribed to those who were allergic to non-steroidal anti-inflammatory drugs.

The post-surgical rehabilitation program included active and passive joint range-of-motion (ROM) exercises, training in activities of daily living (such as toileting and bathing), muscle strengthening, balance exercises, gait training, stair training, endurance exercises, and patient education. ROM exercises, ADL training, full weight-bearing, and gait training were initiated as early as the first postoperative day. The rehabilitation program was scheduled for 60–120 min a day, six to seven days a week, until hospital discharge.

### 2.3. Study Design and Data Collection

This retrospective cohort study aimed to investigate the effects of obesity on short-term patient-reported pain and functional outcomes after TKA. Basic demographic and clinical data, including sex, age, etiology, diagnosis, comorbidities, preoperative walking status, and daily rehabilitation duration, were extracted from the patients’ medical charts. The original version of the Charlson Comorbidity Index (CCI) was used to assess the severity of the comorbidities [41]. Body height and weight were also measured before surgery. Height was measured in the standing position using a stadiometer. Body weight was measured using an electronic scale. Body mass index (BMI) was calculated as body weight divided by the square of the height (kg/m^2^) [42]. The patients were stratified into one of three groups based on the World Health Organization (WHO) classification of BMI: normal weight (BMI ≤ 24.99 kg/m^2^), overweight (25 ≤ BMI ≤ 29.99 kg/m^2^), and obese (BMI ≥ 30 kg/m^2^) [42]. In addition, the self-administered WOMAC was used to assess pain and function before and 2 and 4 weeks after surgery. 

### 2.4. Outcome Measures

The primary outcome was the WOMAC pain and functional scores [43]. The WOMAC is a self-administered patient-reported outcome measure widely used following knee arthroplasty [20]. It has been validated in Japanese patients undergoing TKA and is based on the WOMAC Version LK 3.0. [44]. It utilizes a 5-point Likert scale (1–5) and contains 22 items covering two dimensions: pain (5 items) and function (17 items). All subscales are recommended to be converted to a 0–100-point scale [45,46]. Thus, each WOMAC score was adjusted, with 100 denoting the worst outcome using the following equations: pain score, [(subscale score − 5)/total possible score] × 100; and function score, [(subscale score − 17)/total possible score] × 100. The minimal clinically important differences (MCIDs) in the WOMAC scores for pain and function after comprehensive rehabilitation in patients with knee OA were 7.09 and 11.25 points (on a scale of 0–100), respectively [47]. The MCIDs after TKA are reportedly 11 and 9 points for pain and function, respectively, on a scale of 0–100 [48].

### 2.5. Statistical Analysis

Categorical data are expressed as numerical values (percentage), parametric data as means (standard deviations), and nonparametric variables as medians (interquartile ranges). The Shapiro–Wilk test was used to assess normality. Levene’s test was used to evaluate the equality of variance. Depending on the variable type, the chi-square test, a one-way analysis of variance (ANOVA), or the Kruskal–Wallis test was performed to compare the baseline characteristics of all patients before surgery. In addition, generalized linear mixed models (GLMMs) were used to assess the association between the BMI-stratified groups and the WOMAC pain and function scores preoperatively, at 2 and 4 weeks after TKA. The models were adjusted for age, sex, etiology [OA vs. rheumatoid arthritis (RA)], and comorbidities (assessed by the CCI). The main effects of time, group, and group–time interactions were assessed. The interaction effect indicated the difference in the score change between the BMI groups over time. The results are presented as estimated marginal means with 95 percent confidence intervals (CIs). Using the output data from our GLMMs, a power analysis simulation was performed using the _R_ package, version 4.3.1 (R Foundation for Statistical Computing, Vienna, Austria), _LME_4 package, version 1.1-34, and _SIMR_ package, version 1.0.7 [49]. Furthermore, depending on the variables, a one-way ANOVA or the Kruskal–Wallis test was conducted to compare changes in the postoperative WOMAC scores in the BMI-stratified groups relating to the preoperative score. The effect size values were interpreted as small (η^2^ = 0.01, r = 0.10), medium (η^2^ = 0.06, r = 0.30), or large (η^2^ = 0.14, r = 0.50) [50]. Statistical significance was set at *p* < 0.05. All statistical analyses were performed using IBM SPSS Statistics, version 29.0 (IBM, Tokyo, Japan), except for the aforementioned power analysis simulation.

### 2.6. Ethical Considerations

The study was conducted according to the Declaration of Helsinki and the Ethical Guidelines for Medical and Health Research Involving Human Subjects. This study was approved by the Ethics Committee of the Ibaraki Prefectural University of Health Sciences (approval no: e422; date of approval: 21 December 2023), which waived the need for written informed consent due to the retrospective nature of the study. However, we maintained the opt-out policy mentioned on our hospital’s webpage, whereby eligible participants could withdraw from the study at any time. 

## 3. Results

### 3.1. Participants

The baseline characteristics of the patients stratified according to BMI are presented in Table 1. A total of 102 patients were enrolled in the study. The median age of all the patients was 75.0 years; 85.3% were women. Based on BMI, 30 (29.4%) patients were allocated to the normal weight group (BMI < 25 kg/m^2^), 49 (48.0%) to the overweight group (25 < BMI < 29.9 kg/m^2^), and 23 (22.6%) to the obese group (BMI ≥ 30 kg/m^2^). Seventeen of the twenty-three patients in the obese group had Class I obesity (30 ≤ BMI < 34.9 kg/m^2^), and six had Class II obesity (35 ≤ BMI < 40 kg/m^2^). None of the patients had Class III obesity (BMI ≤ 40 kg/m^2^). These patients were categorized into the obese group owing to the small number of patients in each subgroup. Age (*p* = 0.068), the proportion of women (*p* = 0.254), etiology (OA vs. RA; *p* = 0.068), comorbidity status (as assessed by the number of comorbidities and the CCI; *p* = 0.653, and 0.196, respectively), preoperative walking status (with or without reliance on an assistive device; *p* = 0.902), and preoperative activities of daily living (ADL) (assessed using the functional independence measure motor and cognition scores; *p* = 0.510, and 0.073, respectively) did not significantly differ among the BMI groups. 

### 3.2. Surgery and Rehabilitation

The durations of surgery and daily rehabilitation are summarized in Table 1. The median surgery durations (in minutes) for the normal weight, overweight, and obese groups were 95.0, 98.0, and 106.0, respectively. Pairwise comparisons with a Bonferroni correction revealed that the duration of surgery in the obese group was significantly longer than that in the overweight (*p* = 0.048) and normal weight groups (*p* = 0.006). The duration of surgery was not significantly different between the normal weight and overweight groups. Regarding the duration of daily rehabilitation, no significant differences were found between the BMI groups.

### 3.3. WOMAC Pain and Function Scores

The mean pain and median function scores at baseline were 40.10 and 26.47, respectively (Table 1). The pain and function scores did not significantly differ among the groups (*p* = 0.727 and 0.277, respectively).

Table 2 presents the WOMAC pain and function scores stratified by BMI groups before and 2 and 4 weeks after surgery. Among the 102 patients, 92 (90.2%) and 90 (88.2%) completed the assessment at 2 and 4 weeks after surgery, respectively. Regarding the pain score, the main effect of time (improvement) was statistically significant at 2 and 4 weeks after surgery (*p* = 0.001 and *p* < 0.001, respectively). Considering the function score, the main effect of time (improvement) was statistically significant at 4 weeks after surgery (*p* < 0.001). The main effect of the group and group-by-time interactions was not statistically significant, indicating that the magnitude of change in the patient-reported outcomes was similar across all BMI groups. 

The results of the simulated power analysis for the GLMM models predicting WOMAC pain and function scores were pain score, time effect (89.4%); group effect (9.3%); function score, time effect (74.0%); and group effect (4.9%). Since the *p*-value for the group-by-time interaction effects was relatively large for both the pain and function scores, no further power analysis simulations were performed.

Table 3 presents the between-group comparison of the changes in the WOMAC pain and function scores relating to the preoperative score at 2 and 4 weeks post-surgery. No significant differences were found in the WOMAC pain and function score changes among the BMI groups at each postoperative interval. In addition, a relatively small effect size was observed for each analysis.

## 4. Discussion

The association between obesity and short-term patient-reported outcomes after TKA remains unclear. To the best of our knowledge, this was the first study to report an association between obesity and the early recovery phase with respect to the patient-reported pain and functional outcomes after TKA in Japanese patients. Our findings culminated in two main observations: first, the change in the pain and functional outcomes was similar across all BMI groups at 2 and 4 weeks after TKA. Second, all patients statistically and clinically exhibited relevant pain alleviation and functional improvement shortly after TKA. 

Previous studies exploring the association between obesity and patient-reported outcomes following TKA have indicated conflicting results [16,18,19,20,23,26,27,28,29,30,31,32]. Baum et al. investigated the impact of obesity on patient-reported outcomes 8 weeks after TKA in Germany. They reported that patients with obesity had poorer postoperative pain and function than did those without obesity [30]. Van Egmond et al. assessed these outcomes 6 and 12 weeks after TKA and reported similar results [23]. Both studies identified obesity as a risk factor for poor short-term patient-reported outcomes. However, our results contradicted these findings, and the pain and functional outcomes were similar across all BMI groups 2 and 4 weeks after TKA. This similarity aligned with Papakostidou et al., who reported no significant differences in postoperative WOMAC pain and function scores between those with and without obesity 6 and 48 weeks after TKA in Greece [18]. In addition, Maniar et al. reported a similar functional recovery in patients with Class I and Class II obesity relating to those without obesity 3 months post-TKA in India [32]. 

Considering the clinical benefits of surgery, focusing on score changes rather than simply comparing the postoperative data cross-sectionally is essential, as the latter may reflect differences in preoperative scores [31]. In line with our study, Baghbani-Naghadehi et al. revealed that changes in WOMAC function scores were not significantly different among all BMI groups 3 months post-surgery [20]. Nevertheless, in contrast with our findings, the changes in the WOMAC pain score (pain relief) were found to be more significant in patients in the higher BMI groups (BMI ≥ 35 kg/m^2^) than in those without obesity 3 months post-TKA.

The differences between these studies may be linked to baseline patient characteristics and overall health status [20,26,51]. Our patients were much older, less obese, and had fewer comorbidities than did those in previous studies (i.e., age of 75.0 years vs. mid to late sixties; frequency of patients with BMI ≥ 30 kg/m^2^: 23% vs. 42–65%) [18,19,20,30]. One possible explanation for this variance is the difference in the prevalence of obesity, which is lower in the general Japanese population compared with that in Western countries [10,35,36,37]. In addition, the preoperative WOMAC scores in our study were considerably lower (indicating lesser severity) than those reported by previous studies. The adjusted WOMAC pain and function scores at baseline in our study population were 40.10 and 26.47, respectively. In contrast, the adjusted WOMAC pain and function scores at baseline in a Canadian study were 55.3 and 56.3, respectively [20]. These differences could be attributed to cultural and lifestyle differences and the differences in the indications for TKA established by the healthcare systems of various countries [20]. Japanese people spend more time sitting or kneeling on the floor in pursuit of ADLs such as eating, toileting, and bathing. Hence, Japanese people may experience more difficulties in ADLs and be candidates for TKA at substantially lower WOMAC scores compared with patients in other countries. Furthermore, variations in the duration of the follow-up period and study design (i.e., controlling for confounding factors such as sex and age) may account for the discrepancies in the results [26]. 

Despite these discrepancies, studies have shown that patients derive comparable benefits from TKA, irrespective of their BMI status. Van Egmond et al. assessed patient-reported outcomes using the Knee Disability and Osteoarthritis Outcome Score Physical Function Short-Form and observed clinically relevant improvements 6 weeks after surgery [23]. In line with this, a significant improvement in the WOMAC scores was observed 4 weeks after TKA. Furthermore, this change exceeded the MCID values, implying clinical relevance [47,48]. Although the magnitude of the change was considerably lower than that previously reported during a more extended postoperative period, our results demonstrated that clinically relevant changes might become evident at a much earlier postoperative period compared with that previously reported [20]. 

Inpatient rehabilitation following TKA has been reported to be less efficient in patients with obesity than in those without obesity [52]. In addition, depending on the BMI category, the recovery trajectory may vary by up to three months after TKA [19,20]. Although differences in ethnicity and baseline patient characteristics must be considered, our data illustrated that the BMI category may not have influenced early post-surgical recovery by up to 4 weeks. Moreover, all patients statistically and clinically experienced relevant pain reduction and functional improvement 4 weeks after TKA. This aspect is valuable when surgeons consider the potential benefits of TKA in patients with obesity, despite their reluctance to lose weight, since delaying surgery may have a detrimental effect on pain, dysfunction, psychological well-being, and quality of life [20,53]. In addition, this helps surgeons and healthcare professionals discuss pain alleviation and functional improvement expectations with their TKA candidates [19,20]. Furthermore, this helps rehabilitation therapists formulate rehabilitation programs, and patients maintain a high motivation during the postoperative rehabilitation process. More research is needed to establish conclusive findings and optimize patient satisfaction after TKA. 

This study had some limitations. First, this retrospective cohort study was conducted at a single local hospital in Japan with a relatively small sample size, limiting the generalizability of our results. Second, this study included patients who underwent TKA during the coronavirus disease 2019 (COVID-19) pandemic, which may have impacted rehabilitation progress and short-term patient-reported outcomes. However, we provided continuous inpatient rehabilitation services during this period; the mean duration of daily rehabilitation (hours) across all BMI groups was similar. Thus, the effect of the COVID-19 pandemic might have been minimal. Third, since a relatively small number of patients were classified into the BMI Class I to III obesity subgroups based on the WHO classification of BMI [42], all three classes of obesity were considered as one group for the analysis. This grouping might have limited our understanding of the relationship between higher BMI ranges and patient-reported outcomes. However, since the prevalence of obesity (BMI ≥ 30 kg/m^2^) is relatively low in the Japanese population [35,36,37], we suggest that our results still provide valuable insights for patients in Japan, as well as other Asian countries. 

This study assessed the WOMAC pain and function scores as patient-reported outcomes. However, studies have suggested that patient-reported outcome measures may not accurately reflect objectively assessed functional outcomes [22]. Hence, future studies should explore the association between obesity and objective functional measures, such as knee range of motion, strength, and gait speed. These findings might be necessary for optimizing early postoperative rehabilitation and a clinical care pathway for patients with various obesity statuses. In addition, categorizing obesity by BMI may not accurately reflect excessive adipose tissue accumulation, particularly in those with sarcopenia, an aging-related loss of muscle mass and strength or function [54]. Thus, investigating the association between body composition (i.e., differences in lean and adipose tissue mass) and functional outcome measures might provide knowledge about the quality of care after TKA, as higher numbers of older patients are undergoing this surgery. 

## 5. Conclusions

Statistically significant and clinically relevant improvements were observed in patient-reported pain and functional outcomes in the first 4 weeks after TKA. Furthermore, patients reported comparable benefits, irrespective of their obesity statuses. These data may help healthcare professionals discuss expectations of pain amelioration and functional improvement with TKA candidates, formulate strategies for rehabilitation, and improve post-surgical quality of care. More research is needed to explore the association between obesity and short-term functional outcomes, particularly in Asian populations. 

## Figures and Tables

**Table 1:** Baseline characteristics of all patients.

	Total (*n* = 102)		Normal Weight (*n* = 30)		Overweight (*n* = 49)		Obese (*n* = 23)		*p*
Age	75.00	(71.75, 78.00)	77.00	(72.00, 79.25)	75.00	(73.00, 77.50)	73.00	(68.00, 77.00)	0.068 b
Sex, number of women	87	85.29%	24	80%	41	83.67%	22	95.65%	0.254 c
BMI (kg/m^2^)	27.37	±4.11	23.39	(22.05, 24.26)	27.16	(26.09, 28.75)	31.63	(30.83, 36.05)	<0.001 b *
Etiology, frequency of OA/RA	94/8	92.16%/7.84%	25/5	83.33%/16.57%	46/3	93.88%/6.12%	23/0	100%	0.068 c
Number of comorbidities	2.00	(1.00, 3.00)	2.00	(1.00, 3.00)	2.00	(1.50, 3.00)	2.00	(1.00, 3.00)	0.653 b
CCI	1.00	(0.00, 1.00)	1.00	(0.00, 1.00)	1.00	(0.00, 1.00)	0.00	(0.00, 1.00)	0.196 b
Preoperative walking status									
Self-reliant	66	64.71%	20	66.67%	32	65.31%	14	60.87%	0.902 c
Reliance on assistive devices	36	35.29%	10	33.33%	17	34.69%	9	39.13%	
FIM motor score	85.00	(79.75, 88.00)	85.00	(81.50, 88.00)	85.00	(79.00, 89.00)	83.00	(79.00, 87.00)	0.510 b
FIM cognition score	35.00	(35.00, 35.00)	35.00	(33.75, 35.00)	35.00	(34.00, 35.00)	35.00	(35.00, 35.00)	0.073 b
WOMAC pain score †	40.10	±19.47	42.50	±20.67	39.18	±18.10	38.91	±21.26	0.727 a
WOMAC functional score †	26.47	(16.18, 38.60)	30.88	(19.85, 41.18)	19.12	(16.18, 37.50)	27.94	(17.65, 50.00)	0.277 b
Duration of surgery (minutes)	99.50	(90.00, 101.25)	95.00	(85.50, 106.00)	98.00	(90.00, 109.50)	106.00	(100.00, 127.00)	0.007 b
Duration of daily rehabilitation (hours)	1.66	(1.52, 1.84)	1.66	±0.21	1.65	±0.24	1.66	±0.18	1.000 b

The values presented are *n*, mean ± SD, or median (IQR) for categorical data, parametric data, and nonparametric variables, respectively. Abbreviations: BMI: body mass index; OA: osteoarthritis; RA: rheumatoid arthritis; CCI: Charlson Comorbidity Index; FIM: Functional Independence Measure; WOMAC: Western Ontario and McMaster Universities Osteoarthritis Index; *n*: number; SD: standard deviation; IQR: interquartile range. Normal weight: BMI ≤ 24.99 kg/m^2^, overweight: 25 ≤ BMI ≤ 29.99 kg/m^2^, and obese: BMI ≥ 30 kg/m^2^. a: one-way ANOVA, b: Kruskal–Wallis test, c: chi-square test. * *p* < 0.05. † Scales from 0 to 100, with 100 denoting the worst pain/function.

**Table 2:** WOMAC pain and function scores stratified by BMI before and 2 and 4 weeks after TKA.

		Normal Weight			Overweight			Obese		
WOMAC		*n*	Estimated Mean	(95% CI)	*n*	Estimated Mean	(95% CI)	*n*	Estimated Mean	(95% CI)
Pain †	Pre	30	40.85	(33.16, 48.53)	49	36.83	(29.59, 44.08)	23	35.22	(25.53, 44.91)
	2w	25	28.15	(19.90, 36.40)	46	26.67	(19.35, 33.99)	21	24.30	(14.38, 34.21)
	4w	24	21.64	(13.28, 30.00)	45	17.92	(10.55, 25.29)	21	14.54	(4.62, 24.45)
	Time effect (with respect to preoperative score): 2w, *p* = 0.001 *; 4w, *p* < 0.001 *.									
	Group effect (relating to normal weight group): Overweight, *p* = 0.344; Obese, *p* = 0.280.									
	Group-by-time interaction effect (relating to normal weight group by preoperative score):									
	Overweight × 2w, *p* = 0.603; Obese × 2w, *p* = 0.953.									
	Overweight × 4w, *p* = 0.760; Obese × 4w, *p* = 0.800.									
		**Normal weight**			**Overweight**			**Obese**		
**WOMAC**		*n*	**Estimated** **mean**	**(95% CI)**	*n*	**Estimated** **mean**	**(95% CI)**	*n*	**Estimated** **mean**	**(95% CI)**
Function †	Pre	30	28.34	(22.17, 34.51)	49	24.13	(18.37, 29.90)	23	30.41	(22.67, 38.15)
	2w	25	24.78	(18.12, 31.45)	46	24.08	(18.25, 29.91)	21	25.35	(17.42, 33.28)
	4w	24	13.34	(6.59, 20.10)	45	11.28	(5.41, 17.16)	21	14.29	(6.35, 22.22)
	Time effect (with respect to preoperative score): 2w, *p* = 0.288; 4w, *p* < 0.001 *.									
	Group effect (relating to normal weight group): Overweight, *p* = 0.221; Obese, *p* = 0.792.									
	Group-by-time interaction effect (relating to normal weight group by preoperative score):									
	Overweight × 2w, *p* = 0.402; Obese × 2w, *p* = 0.611.									
	Overweight × 4w, *p* = 0.763; Obese × 4w, *p* = 0.823.									

The values represent the estimated marginal mean and 95% confidence interval. Abbreviations: WOMAC: Western Ontario and McMaster Universities Osteoarthritis Index; TKA: total knee arthroplasty; BMI: body mass index; CI: confidence interval; 2w: 2 weeks post TKA; 4w: 4 weeks post TKA; *n*: number. Normal weight: BMI ≤ 24.99 kg/m^2^, overweight: 25 ≤ BMI ≤ 29.99 kg/m^2^, obese: BMI ≥ 30 kg/m^2^. Generalized linear mixed models were used for the analysis. The dependent variables were WOMAC pain (Model 1) and function scores (Model 2). The fixed effects included in the models were group, time, group × time, etiology (osteoarthritis vs. rheumatic arthritis), age, sex, and comorbidities (assessed using the Charlson Comorbidity Index). Participants were included as random effects. The goodness-of-fit for Models 1 and 2 was as follows: 23,454 and 2254, respectively, for corrected Akaike’s information criteria (AIC); 2361 and 2261, respectively, for the Bayesian information criteria (BIC); 0.498 and 0.449 for the interclass correlation coefficient (ICC); and 0.387 and 0.310, respectively, for variance explained (R^2^). * *p* < 0.05. † The scale is from 0 to 100, with 100 denoting the worst outcome.

**Table 3:** Between-group comparison of the change in the WOMAC pain and function scores relating to the preoperative score 2 and 4 weeks after TKA.

		Total			Normal Weight			Overweight			Obese				
ΔWOMAC		*n*	Mean or Median	±SD or (IQR)	*n*	Mean or Median	±SD or (IQR)	*n*	Mean or Median	±SD or (IQR)	*n*	Mean or Median	±SD or (IQR)	*p*	η^2^ or r
Pain	2w	92	−10.65	±22.75	25	−12.40	±24.80	46	−9.67	±22.59	21	−10.71	±21.52	0.892 a	0.003
	4w	90	−18.72	±21.22	24	−17.92	±23.63	45	−18.33	±21.32	21	−20.48	±18.90	0.910 a	0.002
Function	2w	92	−1.81	±19.47	25	−2.71	±23.14	46	0.13	±18.07	21	−4.97	±18.10	0.593 a	0.012
	4w	90	−11.76	(−23.90, −1.47)	24	−16.18	(−26.47, 2.21)	45	−10.29	(−21.32, −2.94)	21	−11.76	(−28.68, −2.94)	0.769 b	0.056

The values presented are *n*, mean ± SD, or median (IQR) for categorical data, parametric data, and nonparametric variables, respectively. Abbreviations: Δ: delta indicating the change; SD: standard deviation; IQR: interquartile range; η^2^: eta squared; r: effect size; WOMAC: Western Ontario and McMaster Universities Osteoarthritis Index; TKA: total knee arthroplasty; *n*: number. Normal weight: BMI ≤ 24.99 kg/m^2^, overweight: 25 ≤ BMI ≤ 29.99 kg/m^2^, obese: BMI ≥ 30 kg/m^2^. a: one-way ANOVA, b: Kruskal–Wallis test.

## Data Availability

No new data were created or analyzed in this study. Data sharing does not apply to this article.
